# Proximal Association of Land Management Preferences: Evidence from Family Forest Owners

**DOI:** 10.1371/journal.pone.0169667

**Published:** 2017-01-06

**Authors:** Francisco X. Aguilar, Zhen Cai, Brett Butler

**Affiliations:** 1 The School of Natural Resources, University of Missouri, Columbia, Missouri, United States of America; 2 The Center for Agroforestry, University of Missouri, Columbia, Missouri, United States of America; 3 Forest Inventory and Analysis, Forest Service, United States Department of Agriculture, Amherst, Massachusetts, United States of America; Peking University, CHINA

## Abstract

Individual behavior is influenced by factors intrinsic to the decision-maker but also associated with other individuals and their ownerships with such relationship intensified by geographic proximity. The land management literature is scarce in the spatially integrated analysis of biophysical and socio-economic data. Localized land management decisions are likely driven by spatially-explicit but often unobserved resource conditions, influenced by an individual’s own characteristics, proximal lands and fellow owners. This study examined stated choices over the management of family-owned forests as an example of a resource that captures strong pecuniary and non-pecuniary values with identifiable decision makers. An autoregressive model controlled for spatially autocorrelated willingness-to-harvest (WTH) responses using a sample of residential and absentee family forest owners from the U.S. State of Missouri. WTH responses were largely explained by affective, cognitive and experience variables including timber production objectives and past harvest experience. Demographic variables, including income and age, were associated with WTH and helped define socially-proximal groups. The group of closest identity was comprised of resident males over 55 years of age with annual income of at least $50,000. Spatially-explicit models showed that indirect impacts, capturing spillover associations, on average accounted for 14% of total marginal impacts among statistically significant explanatory variables. We argue that not all proximal family forest owners are equal and owners-in-absentia have discernible differences in WTH preferences with important implications for public policy and future research.

## Introduction

An individual’s behavior expressed through choices is influenced by factors intrinsic to the decision-maker and likely associated with others’. The Theory of Planned Behavior [1,2] posits that choices are driven by personal attitudes and norms framed by perceived behavioral controls. Personal attitudes toward a behavior are shaped by different types of information including affective, cognitive, and past behavior and experiences [3]. Norms, often subjective but also formalized through social institutions, can take a descriptive or injunctive form to define prevalent norms or denote what behaviors ought or should be, respectively [4]. The degree of influence of subjective norms on an individual’s behavior is a function of her identity and social proximity to others [5]. Social proximity is a result of *social homophily*—the fact that people tend to emulate the behavior of others who are similar to them [6].

Individual choices are influenced by social interactions which can be facilitated and intensified by geographic proximity [7]. Geographic distances define the actual physical closeness between decision makers or objects. Correlated behaviors and bio-physical conditions have for long been known, as noted in Tobler’s first law of geography “everything is related to everything else, but near things are more related than distanced things”. Underlying spatial phenomena such as social interactions and bio-physical processes drive the appearance of spatially autocorrelated information [8]. Geographic proximity facilitates interaction among individuals (including the sharing of information and norms) which is enhanced by a greater likelihood of managing biophysical resources of similar characteristics. For example, adjacent landowners are more likely to have lands of comparable biophysical (e.g. soil, topography, weather patterns), and economic (e.g. access to the market and financial resources) characteristics than more distantly located owners. Bio-physical and socio-economic resources and their spatially-defined processes co-influence attitudes and behavior toward land management [9,10].

The land management literature is scarce in the spatially integrated analysis of biophysical and socio-economic data. To-date the literature has investigated social distances mainly by exploring neighbor or peer-effects through self-reported landowner surveys and to a lesser extent using in-depth interviews. For instance, several studies have examined the importance of social networks in the adoption of new agricultural practices or technology and some have specifically addressed issues to non-agricultural landscapes [11]. Kueper et al. [12] completed five case studies to derive common themes regarding U.S. forest owner peer-to-peer learning. To our knowledge no past study has analyzed spatially-explicit effects associated to landowners’ management preferences and their ownerships, and specifically to forest owner management preferences and there has been little in the way of empirical econometric-based research focusing on the behavior of spatially proximal family forest owners [13]. Some work has emerged to model behavior regarding natural events occurring within an ecological landscape such as wildfires [14] but there is little in terms of data capturing observed or intended interactions among private forest owners [15]. Beyond bio-physical spatial interaction, there is no quantitative research that has explicitly examined social distance effects on choices regarding forest management and their cumulative effects at the landscape level.

This study examined stated choices over the management of family-owned forests as an example of a land-based resource that captures strong pecuniary and non-pecuniary values with identifiable decision makers—as compared to public or communal lands. Forests are a dominant terrestrial ecosystem in the US covering about a third of the country’s lands of which over half are in private ownership. Family-owned forests (forests owned by families, individuals, trusts, estates, family partnerships and other unincorporated groups) account for about 61 percent of all US private forests [16]. A sample of family forest owners (FFOs) was chosen to study spatially-explicit associations for numerous reasons including: (a) FFOs are known for having a variety of (non) pecuniary motivations for forest ownership, (b) the ability to geo-reference the location of owners and their properties, and (c) capacity to control for different geo-referenced socio-economic and geographic factors. We formally incorporated geographic location in an analysis that also controlled for FFOs’ characteristics aimed at denoting social proximity (e.g. demographic information including age, gender, education, income), attitudes toward harvesting (e.g. cognitive and affective attitudes and past experience) and forest characteristics profiling land bio-physical conditions (e.g. ownership size, proximity to markets). An autoregressive spatial econometric model was chosen to help control for unobserved biophysical processes and spillovers effects occurring over a landscape.

## Literature review

Individuals’ choices are affected by their own attitudes, perceived behavioral control, but are also influenced by others through subjective norms [1]. Intentions are strong predictors of actual behavior and the land management literature in particular stresses the importance of attitudinal effects on the adoption of conservation practices [17,18,19,20,21]. Attitudes in turn are shaped by knowledge, affection and past experiences to determine behavioral beliefs. For example, access to technical assistance, peer-to-peer learning, or membership in a landowner cooperative have been reported as effective means to disseminate information and likely influence attitudes among FFOs [22]. Belief that timber harvesting can be beneficial (or detrimental) to forest health and wildlife habitat, among others, has been identified as an important factor behind (un)willingness to harvest forestlands [23,24,25]. Regarding past experiences the importance of past timber harvesting experiences as a statistically significant predictor of future harvesting intentions [23,25,26].

Beyond a decision-maker, fellow landowners can influence decisions through their impacts on cognition or affection through (in)formal knowledge and information spillovers often strengthened by having nearby ownerships. Interactions and similarity in behavioral patterns emerge from the tendency to emulate the behavior of others who are similar to one self [27]. The degree of social proximity and consequent behavioral association are determined by many factors including individuals’ age, education level, income, ethnicity, social class, occupation and attitudes [28]. Landowners’ attitudes toward timber harvesting can be influenced by family, friends and neighbors [25]. Individuals of similar identity co-influence their choices with larger social consequences [29]. These social linkages can be strengthened by geographic proximity of individuals and their ownerships [5]. As homophily captures the concept of socially proximal individuals, natural resources tend to be more alike as they are closer in geographic space. Hence, social preferences toward management are the result of individual preferences taking place within a social system, but also driven by prevalent resource conditions, with all factors interacting in geography. Land management is a prime example of intertwined socio-economic and bio-physical interactions as landowners manage their lands within resource and social constraints and are often willing to cooperate toward greater pecuniary and non-pecuniary benefits [15,30,31,32,33,34,35].

Spatial analysis of land uses has largely developed within two major research thrusts. The first has focused on modeling land changes where a plot, parcel or pixel is the unit of analysis. Biophysical characteristics such as slope and soil type along socio-economic conditions such as type of ownership have been used as co-variates to explain land changes at different geographic scales [36,37,38,39]. Some have applied different methods to correct for spatial dependence in observation units. Nelson and Hellerstein [40] used a multinomial model with spatial effects to correct spatial dependence when examining the impacts of transportation networks on deforestation. Carrion-Flores and Irwin [41] used a probit model with a spatial sampling routine to model spatial correlation in land use conversion patterns. However, for this stream of studies, researchers seldom incorporated both social and economic factors in their models of management behavior (see [42] for an exception). Some [43] have modeled land cover changes in managed forest ecosystems incorporating socio-economic variables including travel distance to markets, land enrolled in a farming cooperative and a measure of ownership size but did not identify actual decision-makers behind land use choices. The second line of research has focused on modeling landowners’ management preferences under the theoretical framework of random utility theory [44]. This research thrust encompasses the use of surveys to study past or intended future choices. For instance, these studies have included whether to adopt conservation programs or continue crop production in agricultural land [45,46]; whether to retain woodlots for aesthetic reasons [47] or harvest timberland for economic benefits [25]. However, exploration of spatial interdependence of individuals’ decisions relying on survey data can be challenging due to imperfect knowledge of the location of the ownerships and the capacity to overlap socio-economic and bio-physical information [48,49]. Moreover, a complicating factor to co-analyze social distances across geographic and social space is the fact that forest owners today typically live farther away from the parcels they own—almost 40% of family owners in the US live over 1 mile (1.6 km) from their forest ownerships [50]. The relatively recent capacity to georeference socio-economic information and land ownerships has allowed the integration of landowner survey data and bio-physical variables [51,52]. Nevertheless, neither study explicitly controlled for potential spatial autocorrelation of their data.

There is little in the way of econometric research focusing on the spatially-explicit effects of proximal owners and ownerships on forest management preferences. Beyond geographic interaction, there is no research that has quantitatively examined spatially-explicit associations of attitudes toward forest management and their cumulative effects at the landscape level. Aguilar [53] examined econometrically the spatial interaction between wood product manufacturers but based on geographic proximity only and did not include any descriptors of social distances. It is important to incorporate a spatially-explicit dimension to land management decisions because data are often obtained from sources using different sample designs, geographic aggregation scales and that neither socio-economic nor bio-physical data collection necessarily match the spatial scale of the phenomenon under study [54]. The scale mismatch and the inherent need to integrate data from various sources can cause spatially dependent and heterogeneous model coefficients [55]. These two reasons may lead to a spatial component of land use data, which has the tendency to deviate from the often-assumed independence of observations [56]. From an econometric perspective, the incorporation of spatial dependence into the prediction of land use choices can improve model’s goodness-of-fit, reduce the probability of omitted variable bias, provide better model coefficient estimates and improve the analysis of marginal effects [57,58,59,60].

## Theoretical Framework

The analysis of how social and spatial proximity influences FFO management preferences is framed within bounded rational choice theory as a decision maker maximizes utility bounded by resource constraints and her decisions can be influenced by interactions with others [61,62]. In a latent class model of management choices forest owners seek to maximize utility when making management decisions [23,63]. Private landowners’ harvest behavior can be studied using a random utility model where utility is not directly observable but it underlies stated choice outcomes [63]. In the data used in this study forest owners were asked for their willingness to engage in a commercial timber harvest. Formally, the binary choice to be willing to harvest, or not, is given by the difference in utilities *U*_1*i*_-*U*_0*i*_ for the *i*th forest owner where *U*_1_ represents the utility derived from timber harvesting and *U*_0_ from not harvesting. A probit model assumes that this difference *y*_i_* = *U*_1*i*_-*U*_0*i*_ follows a normal distribution and the choices made are reflected on the stated willingness-to-harvest (WTH):

$$\begin{array}{ll} WTH_{i}=1, & if\;y_{i}^{*}>0,\\ WTH_{i}=0, & if\;y_{i}^{*}\leq 0. \end{array}$$ (1)

In this type of latent regression model *y*_*i*_ is modeled as a function of explanatory variables (plus an intercept) captured in *x*_*i*_' with corresponding β coefficients and a random error (ε_*i*_):

$$y_{i}^{*}=x_{i}^{\prime }\beta +\varepsilon_{i}$$ (2)

But the *i*th forest owner’s choice can be affected by neighbors’ harvest preferences [64,65,66]. This dynamic was captured through a spatial autoregressive process where *y*_i_* is a function of neighboring observations and their degree of association captured through a spatial weight matrix *W* as denoted by:

$$y_{i}^{*}=\;\rho Wy_{i}^{*}+x_{i}^{\prime }\beta +\varepsilon_{i},$$ (3)

where *ρ* is a parameter capturing the level of spatial autocorrelation in WTH and *W* is a *n*×*n* spatial dependence matrix that defines the geographic proximity between forest ownerships. Matrix *W* represents weights derived from parcels latitude and longitude coordinates, i.e. observations within shorter distances capture greater weights as they are more likely to be spatially-correlated. In addition to spatial autocorrelation, *ρ* can also capture unobservable latent influences that follow a spatial process [59]. In this study *ρ* represents the strength of observed and latent processes that result in spatially proximal WTH preferences.

## Methods

Research approved by the University of Missouri IRB (project number 1129317). The dataset used in this analysis is included in S1 Dataset.

### Data

The examination of FFOs management choices was empirically conducted by fitting a spatial autoregressive probit model (Eq 2) to stated WTH timber for commercial purposes. Timber harvesting is the most studied behavior of private forest owners [67]. This behavior is of critical importance because harvesting practices have enormous influence on future stand conditions while producing the most tangible forest commodity: wood [13]. Timber is the main source of income derived from continuous forest ownership but non-pecuniary reasons play a very important role on management decisions including whether to harvest and/or convert them to other uses. Consistently, national surveys of US FFOs rank non-extractive reasons (e.g. beauty/scenery, to pass land on to heirs, privacy, nature protection, and part of home/cabin) as key ownership motivations [68]. Moreover, expected revenues from commercial harvesting as a timberland ownership objective can be complementary and instrumental to the attainment of non-pecuniary aims [23].

WTH timber was, hence, used as an indicator of willingness to engage in active forest management. Stated FFOs’ WTH were derived from a sample from the state of Missouri obtained through a mail survey conducted in 2011 [69]. This sample was chosen due to its reported representativeness of local forest ownership conditions and FFOs [23], our ability to georeference responses to parcels and to distinguish residential from absentee owners. The sample focused on 14 counties where 55% of the forestland in the state are located and targeted owners of at least 20 acres (8.1 ha) of forestland since this is a minimum area to justify a commercial timber harvest [70]. The targeting of private ownerships is justified on the fact that they account for about 56% of all US forestlands and in the area of this particular study represent about 82% of forest ownership acreage [68,71]. As with other FFO surveys in the US, respondents were mostly male (82%) and of older demographic (75% were at least 55 years old). Regarding education levels, around 33% of the respondents stated to have had at least a 4-year college degree. Over 25% of the respondents were absentee owners. In terms of past harvest experience, 45% indicated they have previously harvested their forests. Around 71% of responses in the dataset corresponded to no WTH regardless of timber prices. Moreover, these data allowed geocoding addresses, derive georeferenced information from the US Forest Service Inventory and Analysis (FIA) database on forest conditions and other explanatory variables included in our model specific to forest owners and their lands.

**Fig 1:**
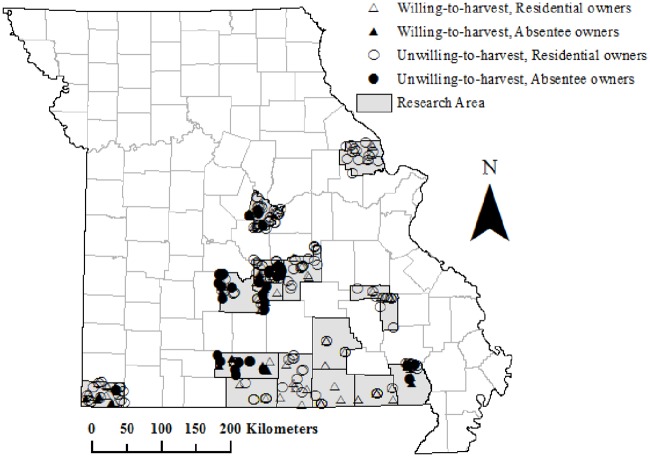
Georeferenced responses distinguishing between residential and absentee owners.

The dataset included 242 survey responses. Two groups were distinguished for econometric analysis within the larger dataset (Table 1). One included all respondents and the second was limited to residential owners only (i.e. excluded absentee FFOs). There were 182 residential owners and 60 absentee owners. Respondents’ parcel locations and corresponding WTH choices are presented in Fig 1. Explanatory variables aimed to control for FFOs’ characteristics to proxy social proximity (demographic information including age gender, education and income levels), attitudes toward harvesting (cognitive and affective attitudes and past harvesting experience) and forest bio-physical characteristics profiling resource conditions (ownership size, proximity to markets, landscape conditions derived from FIA). It is worth noting that in the US there is no regulation at the national (federal) level exclusively affecting forest management [67]. Restriction such as those emerging from the conservation of endangered species can limit timber harvesting but these do not regulate harvesting *per se* instead focusing on habitat conservation. Thus, for this particular research norms affecting forest management behavior are largely limited to subjective norms captured through proximal harvest preferences.

**Table 1:** Variable descriptions and associated descriptive statistics for samples of overall, residential, and absentee-only family forest owners.

Variables	Variable Descriptions	Mean (S.D.)	Mean (S.D.)	Mean (S.D.)	Differences in Sample Means (Residents-only and Absentee only)
		Overall sample (*n* = 242)	Resident-only (*n* = 182)	Absentee-only (*n* = 60)	
**Dependent Variable**					
WTH	Respondents’ stated willingness-to-harvest,:	0.29 (0.46)	0.27 (0.44)	0.35 (0.48)	-0.08
	■ 1 if willing-to-harvest; ■ 0 otherwise				
**Independent Variables**					
Affective, cognitive and past experience					
Beauty***	■ 1 if rated forest ownership “to enjoy beauty or scenery” to be very or extremely important; ■ 0 otherwise	0.68 (0.47)	0.69 (0.47)	0.67 (0.48)	0.02
Privacy***	■ 1 if respondent rated forest ownership “for privacy” to be very or extremely important; ■ 0 otherwise	0.71 (0.45)	0.75 (0.44)	0.60 (0.49)	0.15
Sawlog***	■ 1 if respondent rated forest ownership “for production of sawlogs, pulp-wood or other timber products” to be very or extremely important; ■ 0 otherwise	0.14 (0.35)	0.13 (0.33)	0.20 (0.40)	-0.07
Past harvest experience	■ 1 if respondent had harvested forests before; ■ 0 otherwise	0.45 (0.50)	0.50(0.50)	0.32 (0.47)	0.18
Distance to Service Center***	Distances from forest parcels to the corresponding USDA Service Center	100.90 miles; 162.38 km (41.56)	101.73 miles; /163.72 km (41.40)	98.41 miles; 158.37 km (42.32)	3.32 miles; /5.35 km
Demographics capturing social distances					
Age	■ 1 if respondent was older than 55 years; ■ 0 otherwise	0.75 (0.44)	0.76 (0.43)	0.70 (0.46)	0.06
Gender	■ 1 if male respondent; ■ 0 otherwise	0.82 (0.38)	0.82 (0.38)	0.80 (0.40)	0.02
Education	■ 1 if respondent had at least 4-year college degree; ■ 0 otherwise	0.33 (0.47)	0.29 (0.46)	0.48 (0.50)	-0.17
Income≥50K	■ 1 if respondent’s annual household income was at least $50,000; ■ 0 otherwise	0.45 (0.50)	0.41 (0.49)	0.57 (0.50)	-0.16
Unknown-income	■ 1 if respondent’s annual household income was not reported by respondents; ■ 0 otherwise	0.25 (0.44)	0.26 (0.44)	0.22 (0.42)	0.04
Absentee†	■ 1 if respondent does not live on forest ownership; ■ 0 otherwise	0.25 (0.43)	N/A	N/A	N/A
Land Characteristics					
** **≥500ac (202 hectares)	■ 1 if the number of wood acres owned by respondent is at least 500 acres (202 hectares); ■ 0 otherwise	0.06 (0.24)	0.06 (0.24)	0.07 (0.25)	-0.01
Sawtimber volume	Standardized sawtimber volume in the county where respondents' lands are located (Original unit: cubic feet)	5.21×10^8^ (1.65×10^8^)	5.46×10^8^ (1.62×10^8^)	4.45×10^8^ (1.52×10^8^)	1.01×10^8^
MTNF to parcel location***	Distance from forest parcel to the boundary of Mark Twain National Forest	13.91 miles; 22.39 km (15.27)	15.34 miles; 24.69 km (16.50)	9.57 miles; 15.40 km (9.64)	5.77 miles; /9.29 km
Market accessibility***	Mean distance from forest parcel to the three nearest sawmills	8.63 miles/ 13.89 km (4.98)	8.16 miles/ 13.13 km (5.12)	10.03 miles/ 16.15 km (4.319)	1.87miles; 3.02 km
Market accessibility^2^	Squared mean distance from forest parcels to three nearest sawmills	99.13 miles^2^/ 159.53 km^2^ (96.56)	92.56 miles^2^/ 148.96 km^2^ (95.43)	119.06 miles^2^/ 308.36 km^2^ (98.03)	26.50 miles^2^; 159.40 km^2^

^†^Absentee ownership identifies family forest owners not keeping primary residence within forested parcel.

*** Indicates corresponding means were statistically significant at 1% type-I error level between the overall sample respondents and residential owners.

Georeferenced data included information for the location of forested parcels, US Department of Agriculture (USDA) outreach information centers, sawmills, federally-owned forests, and network of highways. Parcels were georeferenced using Texas A&M Geoservices [72] based on street addresses available through counties’ tax assessor property records or derived directly from maps available online. Location of outreach information centers were geocoded using the street addresses of USDA Service Center locations. These Service Centers provide technical assistance associated with farm and forest management and other services to the public [73]. Location of Service Centers was included to capture FFOs’ potential formal source of information on forest management. Sawmill street addresses were retrieved from the Missouri Department of Conservation [74] to capture accessibility to timber markets. A shapefile of federally-owned forests was also collected to explore differences in management preferences likely associated with proximity to public lands [75]. The most recent Missouri highway network [76] was used to determine geographic distances between FFOs’ forest parcels and outreach information centers (different information centers may serve different counties), sawmills and federally-owned forests using network analyst tool in ArcGIS [77].

### Econometric analysis

Econometrically the standard [Eq 2] and spatial-autoregressive [Eq 3] probit models were estimated using maximum likelihood and a Bayesian approach, respectively. Eq 3 was estimated as follows:

$$y_{i}^{*}=\;\rho Wy_{i}^{*}+x_{i}^{\prime }\beta +\varepsilon_{i},\varepsilon \;\sim \;N\left(0,\sigma_{\varepsilon }^{2}I_{n}\right),$$ (4)

where ε is a random error of zero mean and variance *σ*_*ε*_^*2*^*I*_*n*,_ where *I*_*n*_ is a *n*×*n* identity matrix. The spatial dependence matrix W was derived d first in order to estimate model parameters β. *W* was computationally estimated following a Delaunay triangularization scheme with row standardization [78]. Delaunay triangularization has been used in spatial autoregressive models of land uses to help identify nearby owners [43]. Parcel centroids served as points in a two-dimensional latitude-longitude space with Delaunay neighbors identified as two vertex points of the same triangle. A Delaunay triangulation ensures that no point is inside the circumcircle of every triangle generated from all points in the dataset (Please see [79] for additional procedural details and an illustration). Different from a k nearest-neighbor method that identifies neighbors by a pre-determined number of neighbors, the Delaunay triangulation method captures all potential neighbors that are as close as possible to the parcels. Neighboring relationships were row standardized meaning that the sum of all *w*_*ij*_ values in W of each *y*_*i*_ was equal to unity [80]. It is worth noting that this definition does not imply that neighboring FFOs hold adjacent ownerships although it is possible. Rather the relationship is defined by proximity between georeferenced coordinates. The procedure resulted in a minimum of three and as many as twelve FFOs defined as Delaunay neighbors in the *W* matrix.

Parameters β in Eq 3 were estimated using a Bayesian approach. However, the extension of maximum likelihood methods in the presence of spatial correlation is extremely difficult in binary regressions because autocorrelation patterns produce a likelihood function involving numerous integrals, making direct estimation virtually impossible [81]. Bayesian estimation using Markov-Chain Monte Carlo scheme offers an estimation alternative [80,82]. This Bayesian approach allows for the incorporation of prior distribution information to be used together with information retrieved from collected dataset to make inferences regarding the mean and dispersion of all parameters. Prior probability distribution were set for β ~ *N*(0,1) and *ρ* ~U(0, 1) [59]. The Markov-Chain Monte Carlo scheme was applied to sequentially sample model parameters β, *ρ*, and y* [78]. The number of sampling iterations was set at 20,000 with 5,000 burn-in numbers, to remove bias emerging during initial sampling iterations. Model convergence was tested by running models at least twice with different numbers of draws and further comparing means and variances for the posterior estimates to ensure convergence [59].

Model estimations for the Bayesian spatial autoregressive and standard probit models were conducted using all observations and a reduced dataset of only residential FFOs to examine differences associated with on-site residence as a basic typological descriptor. Hence, we present four model specifications in the Results section. It is worth noting that in our empirical estimation numerous models were used to capture differences in WTH associated with demographic profiles inclusive of the categorization of discrete FFO groups. We identified the use of absentee ownership as a categorization that captured differences in attitudinal variables while maintaining other descriptor information (e.g. attitudes age, gender, income) and refrained from using FFO typologies. As noted by Butler et al. [83] most recent census data for US FFOs offers weak evidence of discrete typological landowner groups, instead suggesting that ownership and management objectives are expressed along an attitudinal continuum.

The impacts of explanatory variables on FFOs’ WTH were examined based on marginal effects, including average direct, indirect and total impacts. Direct impacts captured the average effects of explanatory variables on FFOs’ WTH. Indirect impacts captured the spatial spillover effects brought by the change in explanatory variables to FFOs’ neighbors at the corresponding explanatory variable average values. Total marginal effects aggregated direct and indirect effects. Correctly predicted observations were estimated by comparing observed WTH preferences with predicted probabilities [53]. Standard probit models were also applied to compare estimates with results from the spatial autoregressive models including correctly predicted observations, coefficients magnitude, direction and significance, and marginal effects. Marginal effects in the standard probit models measured the probability change in FFOs’ WTH given one unit change in independent variables at their respective means.

## Results

### Standard Probit and Bayesian Spatial Autoregressive Probit Models

Results of both standard and Bayesian spatial autoregressive probit models are presented in Table 2. Percent of correctly predicted observations across models was about 73–75% which is similar to that reported for the forest industry using the same econometric models [53]. The spatial autoregressive parameter *ρ* was found statistically significant (*p*-value <0.01). The statistical significance of the FFOs’ spatial autoregressive process is consistent with the spatial autocorrelation reported among FFOs’ harvest decisions in France [84]. Values for *ρ* that included all and only residential FFOs were 0.167 and 0.168, respectively. We posit that *ρ* in this particular research denoted evidence of spatially correlated management preferences driven by observed and latent bio-physical and social conditions intensified by their proximity. We propose this interpretation as the spatial weight matrix was derived from georeferenced parcels, hence, the spatial autocorrelation process is likely a function of the geographic proximity between forest ownerships. Our response variable (WTH), is nonetheless a social construct in-turn influenced by biophysical processes at different scales (some at a geographic scale that was not observable, and hence, included in *ρ*). Spillover effects arise from proximal owners and ownerships with FFOs of similar identity and preferences disclosing similar levels of WTH in a bio-physical landscape.

**Table 2:** Results of Standard and Bayesian spatial autoregressive probit models.

Variable	Standard				Bayesian spatial autoregressive			
	All FFOs (Model 1)		Residential FFOs (Model 2)		All FFOs (Model 3)		Residential FFOs (Model 4)	
	Coefficient	*p*-value	Coefficient	*p*-value	Coefficient	*p*-value	Coefficient	*p*-value
Spatial Dependence	N/A	N/A	N/A	N/A	0.167	0.003	0.168	0.003
Affective, cognitive and past experience								
Beauty	-0.058	0.788	-0.130	0.605	-0.009	0.474	-0.036	0.435
Privacy	-0.354	0.121	-0.492	0.090	-0.287	0.074	-0.387	0.057
**Sawlog**	1.076	<0.001	1.101	0.001	1.039	<0.001	1.028	<0.001
Distance to Service Center	<0.001	0.998	-0.002	0.572	-0.002	0.148	-0.004	0.089
**Past harvest experience**	0.736	<0.001	0.838	0.001	0.703	<0.001	0.713	<0.001
Demographics								
**Age**	-0.517	0.020	-0.632	0.021	-0.498	<0.005	-0.534	0.006
Gender (male)	0.001	0.996	0.515	0.153	0.223	0.163	0.577	0.017
Education	0.404	0.052	0.536	0.034	0.119	0.259	0.100	0.319
**Income≥50K**	0.680	0.005	0.827	0.006	0.584	<0.005	0.647	<0.005
Unknown-income	0.207	0.471	0.227	0.514	0.228	0.169	0.237	0.189
Land Characteristics								
≥500acres (202 hectares)	0.117	0.756	0.150	0.737	-0.037	0.451	-0.115	0.371
Sawtimber volume	<0.001	0.785	<0.001	0.965	0.122	0.148	0.138	0.132
MTNF to parcel location	0.007	0.362	0.009	0.314	0.005	0.233	0.005	0.236
Market accessibility	-0.005	0.938	-0.022	0.752	-0.077	0.044	-0.093	0.030
Market accessibility^2^	<0.001	0.905	0.002	0.526	<0.005	0.075	<0.005	0.036
Correctly predicted (%)	75.6		73.6		72.6		72.4	

**Bold** text identifies variables statistically significant 5% type-I error levels across all model specifications.

Across all models four explanatory variables were statistically significant (*p*-value<0.05). Ordered by the value of their respective coefficients these were: owning forests for sawlog production, past harvest experience, income level, and at least 55 years of age. The ownership objective of sawlog production was positively associated with WTH, a result consistent with recent FFO studies [23,85,86]. FFOs’ past harvest experience was also positively associated with WTH. One potential explanation for this effect is that FFOs who had harvested timber previously are more cognizant of the process involved in a commercial harvest, have better market information and understanding of timber harvesting practices than those who have not, else constant. This result is congruent with extant FFO literature (e.g. [25]). Age was found to be negatively associated with WTH as respondents older than 55 were less likely to be willing to harvest timber. These results are also supported by past findings (e.g. [87]) and may be explained by older FFOs on average being more interested in bequest objectives and, thus, less likely to leave potential revenues from standing timber to their heirs [85]. Regarding income it was found that, else constant, FFOs with an annual household income of at least $50,000 were more willing to harvest compared to those with lower stated income. This finding is consistent with results from studies reporting direct and significant impacts of income levels on FFOs’ harvest behavior/preferences (e.g. [85]).

Inclusion of a spatially-explicit autoregressive process yielded discernible differences. For instance, the variable capturing the importance of privacy as an ownership objective was not significant in standard probit models but it exhibited a stronger statistical significance in the Bayesian autoregressive estimation. This shows that after controlling for unobserved spatial effects in *ρ* FFOs who own forests for privacy objectives were significantly less willing to harvest timber. The spatial autoregressive model results show that market accessibility, as denoted by distance to market, had an inverse relationship with WTH indicating that owners of parcels farther away from sawmills systematically exhibited a lower WTH level—although this effect ameliorated with longer distances (as denoted by the sign and significance of the coefficient capturing market distance squared). The modeling of residential-only FFOs’ WTH also showed that male respondents were on average more likely to be willing to harvest timber. This was the case for estimates of both probit models and had a stronger statistical significance in the spatial autoregressive specification. Resident males, all else constant, denoted greater WTH levels than others groups of FFOs.

Results from all models show numerous variables did not significantly affect FFOs’ WTH in our sample. These included distance between forest parcels and USDA Service Center, national forest boundaries, forest parcel size, and county-wide sawtimber volume. Likely the non-significant effect of distance to USDA Service Center is linked to how FFOs obtain information from multiple sources, thus, the geographic proximity to a Service Center had no discernible association. The insignificant impact of distance from forest parcel centroid to the boundaries of MTNF was somewhat unexpected. We had expected that proximity to federally-owned lands would have been linked with higher WTH levels as often nearby private owners have access to better road systems and some may qualify for public-supported cost-share programs that encourage management practices on adjacent private lands. It is likely that some of those dynamics that often occur at a finer geographic scale were simply not captured in this model. If that was the case the effects could have been picked up by *ρ* as noted previously. Likewise, although we attempted to control for a variable denoting sawtimber conditions from FIA county-wide estimates, those are likely to be dominated by conditions within a particular ownership, hence, any likely effects were not detected in this analysis. No detectable effects associated to forest ownership size may be explained by the fact that this particular sample excluded ownerships of less than 20 acres (8.09 hectares). The literature points to differences in ownership objectives that translate into differences in harvesting preferences for larger ownerships (e.g. ≥100 acres/40 hectares) at the regional level [88] but within this sample that was not the case. Lastly, the coefficient capturing the effect of unknown income level had no statistically significant effect on modeled WTH.

### Direct, indirect and total marginal effects

Marginal effects of explanatory variables on WTH are presented in Table 3 distinguishing between standard and spatial-autoregressive probit models Exploration of marginal effects shows that the non-spatial model, on average, slightly over-estimated FFOs’ WTH by approximately 0.3%. Comparison of total marginal effects between the two model and sample specifications show slight overall differences, but the distinction between individual and indirect (spillover) effects is of interest.

**Table 3:** Marginal effects on willingness-to-harvest probability from Standard and Bayesian spatial autoregressive probit models*.

Variable	Standard		Bayesian spatial autoregressive					
	All FFOs (Model 1)	Residential FFOs (Model 2)	All FFOs (Model 3)			Residential FFOs (Model 4)		
	Total	Total	Direct	Indirect	Total	Direct	Indirect	Total
Affective, cognitive and past experience								
Beauty	-0.018	-0.037	-0.002	-0.001	-0.003	-0.010	-0.002	-0.011
Privacy	-0.113	-0.141	-0.086	-0.014	-0.100	-0.102	-0.017	-0.119
**Sawlog**	0.343	0.314	0.294	0.047	0.342	0.270	0.045	0.315
**Past harvest experience**	0.235	0.240	0.186	0.030	0.216	0.188	0.031	0.219
Distance to Service Center	<0.001	-0.001	-0.001	<0.001	-0.001	-0.001	<0.001	-0.001
MTNF to parcel location	0.002	0.002	0.001	<0.001	0.001	0.001	<0.001	0.002
Demographics capturing social distances								
**Age**	-0.165	-0.180	-0.141	-0.023	-0.164	-0.141	-0.023	-0.164
Gender	<0.001	0.147	0.051	0.008	0.060	0.152	0.026	0.178
Education	0.129	0.153	0.041	0.007	0.048	0.026	0.005	0.031
**Income≥500K**	0.217	0.236	0.169	0.027	0.196	0.170	0.028	0.198
Unknown income	0.066	0.065	0.063	0.010	0.073	0.062	0.010	0.073
Land characteristics								
500ac (202 hectares)	0.037	0.043	0.005	0.001	0.005	0.030	0.005	0.035
Sawtimber Volume	<0.001	<0.001	0.027	0.004	0.031	0.036	0.006	0.042
Market accessibility	-0.002	-0.006	-0.017	-0.003	-0.020	-0.024	-0.004	-0.028
Market accessibility^2^	<0.001	<0.001	0.001	<0.001	0.001	0.001	<0.001	0.002

*Marginal effects at continuous means of continuous variables.

Bold text identifies variables statistically significant 5% type-I error levels across all model specifications.

Results of standard probit **Model 1** (residential and absentee respondents included) show that the variable capturing ownership objective of sawlog production had the largest marginal association with WTH. On average, FFOs who reported it as an important ownership objective exhibited a WTH 34.5% greater than those who did not. Past timber harvest experience had a positive marginal effect association with WTH probability of 23.5%. Annual income level of at least $50,000 was associated with a 21.7% greater WTH probability than FFOs of lower income levels. The marginal effects for **Model 2** that included only residential FFOs were similar to those from **Model 1**. The only sizeable noticeable difference in marginal effects between all and residential FFOs was for on the marginal effect associated with gender.

Marginal effects from the spatial model (**Models 3 and 4**) show that on average, indirect effects of statistically significant variables on WTH accounted for 14% of total marginal effects. For instance, the indirect impact from past harvest experience of 0.030 was around 14% of total marginal impact (0.216). This finding stresses how FFOs’ WTH was not only affected directly by changes in explanatory variables but also by subsequent spillovers caused by the autoregressive nature of WTH. Using the same explanatory variable as an example it is also worth noting how indirect marginal effects were greater among the residential-only sample as compared to all FFOs. In the former indirect effects accounted for 14.2% of total effects, while in the latter it was 12.0%. Over a landscape those differences can be substantial. We posit that this result stresses the level of interaction and resulting spillover effects that are more noticeable among residential, hence geographically proximal, FFOs. In **Model 3** (spatial autoregressive model with all respondents) marginal effects of sawlog production ownership objectives were the highest. Its direct marginal effect indicates that the stated WTH probability among FFOs who own forests for sawlog production was on average 29.4% greater than others. The indirect impact of this variable suggests that spillover effects of sawlog production ownership objective were associated with greater WTH probability of 4.7% in the region. In terms of the impacts from FFOs’ other ownership objectives, privacy protection decreased the WTH probability directly by 8.6% and indirectly by 1.4%. Marginal effects of market accessibility variables were ranked the lowest.

Results of the association between WTH and FFO demographic variables provide insights to the likely effects of social proximity. FFOs with annual income of at least $50,000 on average had WTH probabilities 17% higher than those with lower income levels (**Models 3 and 4**). Subsequent spillover effects resulted in an increase of WTH probabilities of 2.7–2.8%. It is again noticeable that higher total marginal effects were found among resident-only respondents and those likely exhibited a greater degree of incidence of indirect marginal effects. We argue that the strength of social proximity effects can be partly discerned from indirect effects. For instance, for an average FFO whose annual income level was at least $50,000, the greater disparity in income levels between neighbors (i.e. neighbors with income less than $50,000) compared with zero income distances (i.e. neighbors of same income level) there was a lower WTH probability of 2.7% (**Model 3**). Direct impact of age group was -0.141 indicating that FFOs who were older than 55 years were 14.1% less likely to harvest timber compared with younger FFOs. Indirect impacts of age suggested social distances between age groups may affect FFOs’ WTH probability by 2.3%. And it is noticeable that indirect marginal effect was greater among resident FFOs. Moreover, gender effects were more sizeable in the resident-only sample and the indirect effect accounted for 14.6% of total effects in the spatial model specification. Direct gender association on WTH probability suggests that male FFOs were 15.2% more likely to harvest timber compared with female counterparts, all else constant. An FFO whose neighboring parcels were owned by male owners was 2.6% more willing-to-harvest timber. This seems a relatively small individual signal but compounding effects over a landscape can be sizeable.

## Discussion

### Spatially autocorrelated management preferences

The literature discussing FFOs observed or stated behavior is ample (e.g. [22]) yet issues related to spatially-explicit interactions and consequent implications to management beyond individual ownerships have remained elusive. Although the context of the data used for this study is limited to the geographic and temporal scope of the original survey, findings can be relevant to other regions of similar ownership profiles and underlying dynamics.

There were several noticeable trends in this analysis of a georeferenced sample of Missouri FFO ownerships. We found strong evidence of spatial autocorrelation in stated WTH among FFOs suggesting that these preferences are not independent random events as often assumed in traditional statistical models. The strength and significance of the coefficient *ρ* suggest that stated WTH was strongly correlated within geographically proximal FF ownerships and this effect was stronger among male resident owners. It is likely that *ρ* also captured effects of unobserved spatially correlated bio-physical variables as could have been the case of explanatory variables exerting an impact on WTH at a scale smaller than included in our model (e.g. county-wide sawtimber estimates as compared to localized parcel conditions). As expected, the estimates for *ρ* were very similar for both samples (all FFOs and resident owners only) as the weights in the spatial weights matrix captured geographic proximity effects. Spatial autocorrelation associated WTH was linked to bio-physical conditions likely stemmed from nearby forested parcels being more similar than others. Results from the spatially-explicit model point to the importance and differences between spill-over effects. Among statistically significant explanatory variables associated indirect effects accounted for about 14% of total effects. Between those variables the magnitude and statistical significance of indirect effects was greater for management objectives that might have important spillover effects (e.g. sawlog harvesting) than benefits accrued within a parcel (e.g. privacy). Findings stress the cumulative indirect effects of WTH with consequences over a landscape well beyond individual ownerships.

It is worth noting that the value of *ρ* can vary depending on conditions specific to the area under study and sample intensity. Reported values for *ρ* ranging from 0.112 to 0.328 depending on areas sampled [43]. Differences in approaches, including the fact that we looked at WTH, as compared to drivers of land clearing [43], prevent direct comparisons with our results (0.167–0.168). In our study average distances from the Delaunay triangulation ranged from 2.6 km to 42.8km. Although our sampling was able to detect spatial autocorrelation, the magnitude and significance of *ρ* might be strengthened or weakened depending on the explanatory variables controlled in the model and the geographic scale examined. This is an empirical issue that deserves further investigation.

WTH responses were largely dominated by affective, cognitive, and past experience covariates as captured through variables including ownership objectives for sawlog production and past harvest experiences. Demographic variables, including income and age dominated the role of social distances on WTH and the case of gender is one that illustrates this well. FFOs of the same gender, thus deemed more socially proximal, exhibited positive and significant marginal effects with relatively large indirect (spillover) impacts. This level of stronger spatial autocorrelation might reflect a greater degree of interaction among socially-proximal landowners, these being resident male FFOs. It is also likely that associations for onsite and absentee FFOs may have different sources. Proximal associations on residential FFOs’ WTH may primarily come from other FFOs living near their forest ownerships. In the case of absentee FFOs’ WTH preferences these may not only be affected by FFO of neighboring parcels but also those living near their residence whether those residential peers are FFOs or not.

### Management implications

The findings have several implications for management, public policy as well as future research. Regarding management, there is a growing concern over parcelization of forested ownership. Ownership parcelization is prevalent throughout US [89]. One of the challenges brought up by parcelization is the capacity to implement forest management practices including commercial harvesting. Forest operations can have large fixed costs that spread over smaller management sites might become cost-prohibitive [90,91]. In turn, financially unfeasible operations of individual ownerships can have cumulative consequences at the landscape level and limit the ability to achieve ecosystem-level objectives. We argue that spatial autocorrelation in WTH preferences could reduce parcelization’s constraint on commercial harvests. If the spillover effects found in this research can translate into effective cooperation the coordination among FFOs to implement management across ownerships might help ameliorate the challenges brought up by parcelization.

Arguably, a public policy that can influence attitudes as major drivers behind WTH will have spillover effects. In this regard, it could be more effective to target socially-proximal FFOs. This research noted that although all FFOs share a common identity as forest owners, not all landowners are equal. Owners-in-absentia might correspond to a different type of FFO group while those of closest social distances were found to be resident males at least 55 years of age of annual income of at least $50,000. This group was identified to be more prone to engage in timber harvesting and had the largest WTH indirect effects. Absentee owners in this sample might be profiled as a FFO type that on average exhibited similar associations between select explanatory variables and WTH but of relative different direct and indirect effects.

The fact that absentee FFOs do not reside on or near their forest ownership suggest that they might be more socially proximal to those living around their primary residences. One potential line of research extending from this should inquire on absentee FFOs. New research efforts should look into surveying individuals residing around absentee FFOs to determine whether similar proximal associations are observed as with residential owners and a different group of peers might be identified. Specifically, research investigating FFO associations might need to delve into actual social networks influencing WTH that might not be exclusive of other FFOs and include individuals who might not have any kind of forest or rural ownership but could well influence FFO management decisions.

## Conclusions

Preferences toward forest management captured through WTH were surveyed from a sample of FFOs, georeferenced to forested parcels, and modeled as a function of selected affective, cognitive, past experiences, demographic information and land characteristics. WTH responses were largely explained by affective, cognitive, and past experience covariates as captured through variables including ownership objectives for sawlog production and past harvest experiences. Demographic variables, including income and age dominated the role of social distances on WTH and helped define groups of peers.

Comparison of standard and spatially-explicit probit models showed that total marginal effects of explanatory variables were similar. Spatially-explicit models determined that indirect effects, capturing spillover association, on average accounted for 14% of total marginal impacts. Distinction between all and residential-only FFOs showed that among the latter gender was another indicator associating social proximity and WTH. Thus, the group of FFOs of closer identity was defined as resident males over 55 years of age of annual income of at least $50,000. FFOs in absentia might be classified as a different group that is expected to grow in size over time. There is a need to better understand absentee FFO associations, in particular with other individuals living near their residences—who may be forest owners or not—but can influence absentee FFO’s management preferences.

Beyond FFO descriptors variables capturing forest ownership characteristics showed a weak level of significance which may suggest that conditions occurring at smaller geographic scales affecting WTH may have also been included in the autoregressive coefficient, strengthening the spatial autocorrelation of observations. Hence, there was little difference in the value of *ρ* coefficients between all and residential-only FFO samples. Nonetheless, market accessibility was found to significantly affect FFOs’ WTH. It is of interest to note that FFOs of ownership located farther sawmills were on average less likely to be willing to harvest and this effect faded over longer distances. The distance from FFO’s parcels, to USDA Service Center and public lands were not influential on WTH.

The findings from this research stress the importance of considering spatially-explicit effects when studying individual owners’ land use choices. As better and more spatially defined information becomes available the detection of spatial effects might be more evident whether through spatial regressive models or explanatory variables defined at the appropriate scale. Individual land management decisions are not random and driven by land conditions but also influenced by individuals’ attitudes and how these are influenced by socially and geographically proximal forest owners. Future survey-based studies should consider georeferencing responses to landowner parcels. Spillover consequences detected through indirect marginal effects associated with socio-economic variables has important public policy implications. Public policy instruments that influence attitudinal conditions will have the greater potential through spillover effects to attain landscape-level objectives.

## Supporting Information

S1 Dataset (XLSX)
